# Racial and ethnic disparities in the perception of respect from physicians among skin cancer patients in the United States

**DOI:** 10.1016/j.jdin.2023.01.009

**Published:** 2023-01-27

**Authors:** Lauren M. Fahmy, Celine M. Schreidah, Larisa J. Geskin

**Affiliations:** a Columbia University Vagelos College of Physicians and Surgeons, New York, New York; b Department of Dermatology, Columbia University Irving Medical Center, New York, New York

**Keywords:** basal cell carcinoma, distrust, ethnic disparities, explaining, listening, melanoma, mistrust, patient perception, patient-physician communication, patient-physician interaction, patient-physician relationship, racial and ethnic disparities, racial disparities, respect, skin cancer, squamous cell carcinoma, aOR, adjusted odds ratio, CI, confidence interval, NHW, non-Hispanic White, NMSCs, non-melanoma skin cancers

## Abstract

**Background:**

Racial and ethnic minority groups are at increased risk of poor skin cancer outcomes. Successful patient-physician communication is linked to better health outcomes, but it is unknown whether disparities in perceived care exist among skin cancer patients.

**Objective:**

To investigate whether there are racial and ethnic disparities in the perception of physicians showing respect, listening, and explaining during clinical encounters.

**Methods:**

A cross-sectional study was conducted using data from participants with a self-reported skin cancer history from the 2008 to 2017 and 2019 Medical Expenditure Panel Survey. Race and ethnicity were self-identified.

**Results:**

Of 5570 participants, 5263 were non-Hispanic White and 307 were racial and ethnic minority individuals. Racial and ethnic minority participants were less likely to report that their doctors show them respect, listen to, and explain to them than non-Hispanic White participants, even when adjusting for age, sex, insurance type, health status, and survey year. Among racial and ethnic minority participants, perceptions of physicians listening and explaining were strongly associated with perceived respect.

**Limitations:**

Lack of disaggregated racial and ethnic subgroup analysis.

**Conclusions:**

Our findings suggest racial and ethnic disparities in perceived care among skin cancer patients. Future research is warranted to determine whether such perceptions contribute to disparities in skin cancer care and/or outcomes.

**Capsule Summary**

• Racial and ethnic minority skin cancer patients were less likely to feel respected, listened to, and explained to during clinical encounters than non-Hispanic White patients.
• Improving patient-centered communication may mitigate disparities in perceived respect from physicians, though further research is needed.

## Introduction

Although melanoma and non-melanoma skin cancers are more common in non-Hispanic White (NHW) individuals, they are associated with disproportionate morbidity and mortality in individuals belonging to racial and ethnic minority groups.^1^^,^^2^ The reasons for these disparities are multifactorial and poorly understood. While many studies have focused on socioeconomic factors, little attention has been paid to disparities in interpersonal aspects of health care as potential drivers of inequity. A growing body of evidence suggests that strong patient-physician relationships engender trust and can positively influence health outcomes.^3^ Racial and ethnic minority patients are more likely to experience lower quality interactions with their physicians which may contribute to health disparities.^4^ Prior studies have shown that non-White patients are less likely to perceive respect from physicians than their NHW counterparts. However, most studies have examined specific populations^5^^,^^6^ and/or use data from over 2 decades ago^7^ which may not fully reflect the shifting racial and ethnic demographics of the U.S. population. Furthermore, to our knowledge, no studies have evaluated racial and ethnic disparities in the quality of patient-physician interactions among skin cancer patients. We used cross-sectional survey data from patients with skin cancer to assess the association between racial and ethnic minority status and patients’ perception of physicians showing respect, a fundamental component of strong patient-physician relationships. We also evaluated patient perceptions of physicians’ ability to listen and explain during clinical encounters and assessed the degree to which these communication skills contributed to the perception of respect.

## Methods

This study included data from the 2008 to 2017 and 2019 Medical Expenditure Panel Survey, a population-based survey of civilian noninstitutionalized individuals that uses a complex sampling scheme.^8^ Data from 2018 were excluded as the primary outcome was not assessed during this year. A subpopulation analysis of participants who reported a history of melanoma and/or non-melanoma skin cancer was performed. The study was exempt from the Columbia University Institutional Review Board because deidentified, publicly available data were used.

The NHW group included individuals who self-reported a White race and non-Hispanic ethnicity. The racial and ethnic minority group included individuals who self-reported a Hispanic ethnicity and/or any of the following races: Black, Asian, Native American, Alaskan Native, or multiracial. Given that skin cancer outcomes are worse among most racial and ethnic minority groups,^1^ a collective group was used in our study. This also allowed us to include all racial and ethnic groups rather than excluding those with small sample sizes.

The primary outcome variable in our study was participants’ responses to “how often doctors show respect.” Responses included “never”, “sometimes”, “usually”, or “always”. Participants who responded “usually” or “always” were considered to perceive that their doctors showed respect. Similarly, secondary outcome variables included responses to “how often doctors listen” and “how often doctors explain.” Covariates included self-reported age, sex, health insurance type, skin cancer type, health status, and survey year.

Sociodemographic and outcome variables were compared between groups using a Pearson χ2 test. Univariable and multivariable logistic regression models were used to compare outcome variables between groups; multivariable models adjusted for age, sex, insurance type, health status, and survey year. All analyses were performed with STATA 17 statistical software (StataCorp). Appropriate sample weights were used to account for the complex survey design and produce nationally representative prevalence estimates.

## Results

Among 5570 individuals reporting a history of skin cancer, 5263 participants were included in the NHW group (weighted percent 96.9%) and 307 were included in the racial and ethnic minority group (3.2%). Compared to the NHW group, the racial and ethnic minority group was more likely to be young, female, and uninsured or publicly insured. Participants in the racial and ethnic minority group were less likely to report doctors showing respect, listening, and explaining compared to participants in the NHW group (Table I). In a multivariable model adjusting for age, sex, insurance type, health status, and survey year, racial and ethnic minority group participants had 44% lower odds of reporting doctors show respect (adjusted odds ratio [aOR]: 0.56, 95% confidence interval [CI]: 0.32-0.99), 36% lower odds of reporting doctors listen (aOR: 0.64, CI: 0.46-0.93), and 53% lower odds of reporting doctors explain to them during clinical encounters (aOR: 0.47, CI: 0.27-0.83) compared to NHW participants (Table II). Among racial and ethnic minority participants, those who reported that their physicians explained (aOR: 22.7, CI: 6.42-80.52) or listened (aOR: 7.20, CI: 1.92-27.10; *P* < .001) had higher odds of reporting that their doctors showed respect (Table III).

**Table I:** Population characteristics by racial and ethnic minority status

Characteristic	Unweighted No. (weighted %)		*P* value
	Non-Hispanic White (*n* = 5263)	Racial and ethnic minority (*n* = 307)	
Age, y			
18-39	202 (3.7)	38 (10.6)	<.001
40-64	2051 (41.9)	125 (43.5)	
65-85	2557 (47.8)	115 (40.4)	
>85	396 (6.6)	25 (5.5)	
Sex, female	2593 (49.4)	169 (60.3)	.020
Racial and ethnic group			
Hispanic (any race)	0 (0)	175 (48.7)	<.001
Non-Hispanic Alaskan Native or American Indian	0 (0)	8 (5.8)	
Non-Hispanic Asian or Pacific Islander	0 (0)	22 (4.5)	
Non-Hispanic Black	0 (0)	52 (12.2)	
Non-Hispanic multiracial	0 (0)	50 (28.8)	
Non-Hispanic White	5263 (100)	0 (0)	
Insurance type			
Private	3725 (74.4)	165 (64.4)	.031
Medicaid or public	1385 (23.1)	125 (30.6)	
Uninsured	153 (2.5)	17 (5.1)	
Skin cancer type			
Non-melanoma	3753 (71.9)	187 (60.5)	.01
Melanoma	1510 (28.1)	120 (39.5)	
Health status, poor	162 (2.8)	21 (4.8)	.056
Perception of respect by physicians	4113 (95.2)	192 (90.7)	.008
Perception of physicians listening	2715 (63.0)	114 (51.4)	.003
Perception of physicians explaining	4114 (95.2)	193 (90.3)	.007

**Table II:** Multivariable logistic regression model∗ of factors associated with the perception of physician showing respect, listening, and explaining during clinical encounters

Characteristic	Perception of physicians showing respect		Perception of physicians listening		Perception of physicians explaining	
	aOR (95% CI)	*P* value	aOR (95% CI)	*P* value	aOR (95% CI)	*P* value
Racial and ethnic minority status						
NHW	1 [Reference]		1 [Reference]		1 [Reference]	
Racial and ethnic minority	0.56 (0.32-0.99)	.047	0.64 (0.46-0.93)	.017	0.47 (0.27-0.83)	.009
Age, y						
18-39	1 [Reference]		1 [Reference]		1 [Reference]	
40-64	1.14 (0.55-2.37)	.72	0.93 (0.61-1.41)	.73	0.63 (0.22-1.76)	.38
65-85	1.72 (0.85-3.49)	.13	1.12 (0.75-1.70)	.57	0.84 (0.31-2.28)	.73
>85	2.54 (1.06-6.07)	.037	1.15 (0.71-1.87)	.57	0.45 (0.16-1.29)	.138
Sex, female	1.14 (0.86-1.51)	.35	0.93 (0.79-1.09)	.35	0.85 (0.60-1.21)	.38
Insurance type						
Private	1 [Reference]		1 [Reference]		1 [Reference]	
Medicaid or public	0.48 (0.31-0.73)	.001	0.82 (0.67-1.00)	.06	0.59 (0.38-0.91)	.018
Uninsured	0.46 (0.20-1.07)	.072	0.96 (0.54-1.70)	.90	0.57 (0.20-1.62)	.29
Health status, poor	0.33 (0.19-0.60)	<.001	0.44 (0.31-0.63)	<.001	0.39 (0.22-0.69)	.001

*aOR*, Adjusted odds ratio; *CI*, confidence interval; *NHW*, non-Hispanic White.

∗ Adjusted for age, sex, insurance type, and survey year.

**Table III:** Univariable logistic regression model of factors associated with the perception of physician showing respect among racial and ethnic minority patients with skin cancer

Characteristic	aOR (95% CI)	*P* value
Perception of physicians listening	7.20 (1.92-27.10)	.004
Perception of physicians explaining	22.73 (6.42-80.52)	<.001

*aOR*, Adjusted odds ratio; *CI*, confidence interval.

## Discussion

In this nationally representative, cross-sectional study, skin cancer patients in racial and ethnic minority groups were less likely to feel that their doctors respect, listen to, and explain to them compared to NHW patients. These perceptions persisted when adjusting for sociodemographic factors including age, sex, insurance status, and health status. To our knowledge, this is the first study examining racial and ethnic disparities in skin cancer patients’ perceptions of respect and other measures of patient-physician relationships. Our results revealing such disparities are consistent with prior studies in other patient populations (eg, in primary care settings),^5^, ^6^, ^7^ underscoring the systemic and persistent nature of this problem in medicine, and emphasizing the need to understand and address the underlying causes of these disparities.

Several studies have shown a correlation between patient-physician communication and health outcomes.^7^^,^^9^, ^10^, ^11^, ^12^ Research has also begun to explore the mechanisms by which health care encounters influence disparities in the use of health care services or quality of care. For example, patients who feel disrespected by their physicians are less likely to seek care, adhere to treatment recommendations, and attend follow-up appointments. Considering that optimal care for skin cancer patients includes prompt diagnosis, expeditious treatment, and long-term surveillance and/or regular skin examinations, it is possible that clinical care at any of these points may be compromised if a patient feels disrespected by their physician. Although we cannot draw conclusions about the impact of our findings on clinical outcomes, we hypothesize that racial and ethnic disparities in patient perceptions of respect may influence health care utilization and contribute to disparities in skin cancer morbidity and mortality. Future studies are needed to establish this link.

The disparities identified in our study are likely driven by multiple complex factors including bias among health care providers, language barriers, and patient mistrust rooted in centuries of racial discrimination and exploitation in medical settings.^13^^,^^14^ As such, addressing these disparities is not a simple or straightforward task and will likely require changes to broader organizational structures in the form of policies, laws, and procedures that promote equity in the U.S. health care system and beyond. Nonetheless, our study highlights one practical takeaway; among racial and ethnic minority patients, patient perceptions of physicians listening and explaining were strongly associated with feeling respected by physicians. This suggests that improving patient-centered communication with racial and ethnic minority patients may be an intervention that helps mitigate the observed disparity in perceived respect. A recent qualitative study examining patient perspectives on how to demonstrate respect in a primary care setting provides insight into various strategies that clinicians can utilize. Examples include using interpreters for patients speaking another language, prompt communication (eg, regarding appointment delays, following up on results), considering how finances affect patients’ decisions, and ensuring continuity of care.^15^ Future research should explore racial and ethnic minority skin cancer patients’ perspectives on barriers to effective communication to develop targeted interventions that address the unique needs of this population.

Our study has several important limitations. We defined our study population using self-reported skin cancer history which may be subjected to recall bias. The number of participants in the racial and ethnic minority group was limited, and further disaggregated analysis of racial and ethnic subgroups was not possible due to small sample sizes. When asking participants about their perceptions, survey questionnaires did not specify a certain type of physician. Therefore, our results are not specific to dermatologists but apply to all types of physicians. Because patients with a personal history of skin cancer are recommended to undergo routine skin cancer screening examinations, many, if not all, of the patients included in our study should have been seeing dermatologists on a regular basis. In addition, given that negative health care experiences influence subsequent trust in and engagement with the health care system,^7^^,^^16^ negative experiences with other providers may impact patients’ skin cancer care (eg, leading to delays in seeking care, reduced likelihood of following up on abnormal biopsy results, etc.). It is possible that racial and ethnic minority patients, who more commonly experience medical trauma,^17^ are more likely to perceive their physicians as showing a lack of respect in general as a result of past experiences with the health care system. In this case, strategies that address the underlying causes of medical mistrust may be more important than a specific focus on demonstrating respect, although these concepts are bioethically related, and therefore, unlikely to be fully independent from one another.^18^, ^19^, ^20^ Because our study assessed patient perceptions, we cannot comment on whether our findings reflect actual differences in provider communication. However, patient perceptions have a greater impact on outcomes than physician behavior,^11^ underscoring the importance of our findings irrespective of objective measures of physician communication.

## Conflicts of interest

L.J.G has served as an investigator for and/or received research support from Helsinn Group, J&J, Mallinckrodt, Kyowa Kirin, Soligenix, Innate, Merck, BMS, and Stratpharma; on the speakers’ bureau for Helsinn Group and J&J; and on the scientific advisory board for Helsinn Group, J&J, Mallinckrodt, Sanofi, Regeneron, and Kyowa Kirin. L.M.F and C.M.S have no conflicts of interest to disclose.
